# Preparation and Characterization of High-Density Polyethylene with Alternating Lamellar Stems Using Molecular Dynamics Simulations

**DOI:** 10.3390/polym16020304

**Published:** 2024-01-22

**Authors:** Mohammed Althaf Hussain, Takashi Yamamoto, Syed Farooq Adil, Shigeru Yao

**Affiliations:** 1 Central Research Institute, Fukuoka University, Fukuoka 814-0180, Japan; 2 Graduate School of Science and Engineering, Yamaguchi University, Yamaguchi 753-8512, Japan; 3 Department of Chemistry, College of Science, King Saud University, P.O. Box 2455, Riyadh 11451, Saudi Arabia; sfadil@ksu.edu.sa

**Keywords:** HDPE model, MD simulations, crystallinity, mechanical property, plastic recycling, plastic properties, entanglements

## Abstract

Mechanical recycling is the most efficient way to reduce plastic pollution due to its ability to maintain the intrinsic properties of plastics as well as provide economic benefits involved in other types of recycling. On the other hand, molecular dynamics (MD) simulations provide key insights into structural deformation, lamellar crystalline axis (c-axis) orientations, and reorganization, which are essential for understanding plastic behavior during structural deformations. To simulate the influence of structural deformations in high-density polyethylene (HDPE) during mechanical recycling while paying attention to obtaining an alternate lamellar orientation, the authors examine a specific way of preparing stacked lamella-oriented HDPE united atom (UA) models, starting from a single 1000 UA (C_1000_) chain of crystalline conformations and then packing such chain conformations into 2-chain, 10-chain, 15-chain, and 20-chain semi-crystalline models. The 2-chain, 10-chain, and 15-chain models yielded HDPE microstructures with the desired alternating lamellar orientations and entangled amorphous segments. On the other hand, the 20-chain model displayed multi-nucleus crystal growth instead of the lamellar-stack orientation. Structural characterization using a one-dimensional density profile and local order parameter {*P*_2_(*r*)} analyses demonstrated lamellar-stack orientation formation. All semi-crystalline models displayed the total density (ρ) and degree of crystallinity (χ) range of 0.90–0.94 g/cm^−3^ and ≥42–45%, respectively. A notable stress yield (σ_yield) ≈ 100–120 MPa and a superior elongation at break (ε_break) ~250% was observed under uniaxial strain deformation along the lamellar-stack orientation. Similarly, during the MD simulations, the microstructure phase change represented the average number of entanglements per chain (<*Z*>). From the present study, it can be recommended that the 10-chain alternate lamellar-stack orientation model is the most reliable miniature model for HDPE that can mimic industrially relevant plastic behavior in various conditions.

## 1. Introduction 

Thermoplastic high-density polyethylene (HDPE) is a polymer that is used daily. Nevertheless, the accumulation of plastic waste is a global issue [1,2]. Hence, recycling is the best way to thwart environmental plastic pollution. Among various recycling methods, mechanical recycling, due to its retention of intrinsic properties and energy efficiency, appears to be superior to other popular recycling methods, such as chemical recycling and pyrolysis [3,4]. Recent twin-screw experiments by Okubo et al. proposed the optimized conditions for the mechanical recycling of thermally degraded HDPE [5]. The authors claimed that the twin-screw extruder (TSE) equipped with a molten resin reservoir (MRR) allows the structural relaxation of the HDPE chains, improving the chain compatibility and enhancing the recycled HDPE’s ductility. To further enhance/predict the processing conditions for mechanical recycling, the plastic’s microstructure behavior in a molten state and MRR process conditions must be explored at the molecular level using molecular dynamics (MD) simulations. 

Molecular simulation requires an appropriate semi-crystalline model that mimics bulk behavior through which numerical analysis and analytically solving the equations of motion can be carried out. In addition to this, advancements in MD simulation, which can document the molecular chain behavior over a millisecond time scale provided for polymers represented either as a united atom (UA) or as coarse-grained (CG) models, are indispensable for preparing a simple, more affordable HDPE semi-crystalline model structure comprising random or zigzag amorphous regions and ordered crystalline lamellar segments [6,7,8,9,10,11]. By taking advancements of UA or CG modeling in MD simulations, the macroscopic mechanical recycling methods would be simulated to predict the microstructure–mechanical property behavior [6].

To resemble the true nature of the HDPE, the density range of the simulated model must be ~0.94–0.97 g/cm^−3^, in which the density 0.85 g/cm^−3^ represented the amorphous part of the models, and while ~1.0 g/cm^−3^ represented the ordered crystalline lamellar stems density. The degree of HDPE’s crystallinity (χ) in experiments was ~70%, and so was the computational model [7]. In addition, even with simple ethylene units, plastics also have unique topological entanglements from long chains that must also be a part of the simulated models [8]. Finally, while supporting a molecular cavitation mechanism, the tensile test must accomplish the stress–strain curve (SSC) features, including elasticity, yield point, plasticity behavior, and chain hardening [7]. Despite MD’s principal role and relevance in understanding the molecular mechanism of plastics, the literature reports have been scarce, since concocting the semi-crystalline structure model with both amorphous and crystalline segments within the box is challenging, and many new strategies have been implemented to date; however, they have provided limited information on a simple and robust procedure that could be applied to all semi-crystalline structures [7,12].

Fortunately, MD simulations have been well-recognized for such model building and applications involving structural deformation to a more significant extent. Various reports support isothermal cooling from the molten state when preparing a semi-crystalline model [7,12,13,14,15,16,17]. In isothermal crystallization, the packed polymers were melted to a high temperature, much above their melting point (MP). An isotropic melt structure would be prepared through equilibration, further cooling down to room temperature (RT ~300 K) in a subsequent step using distinctive quenching rates. Finally, a prolonged cooling (>100 ns MD simulation time length) near 300 K must be performed until the ordered chain lamella is obtained in the microstructure to discriminate both crystalline and amorphous phases. The isothermal crystallization of HDPE is widely studied using the full atomistic and coarse-grained model parameters, and the molecular mechanism of the mechanical properties was also revealed [7,18,19,20]. Conversely, uniaxial or biaxial stretched crystallization to prepare the semi-crystalline models has been shown in the literature [20]. Yamamoto’s pioneering work thoroughly discusses the crystal network mechanism in the uniaxially extended semi-crystalline models [12]. Polyethylene’s melt memory effects on its recrystallization and lamellar-stack orientation-dependent mechanical responses were investigated using the uniaxially stretched models. The microstructure-dependent mechanical property relationship has been well estimated from the interphase Monte Carlo (IMC) simulated lamellar stacked models by Rutledge et al. [21,22]. The lamellar stack model has been constructed with respective densities of amorphous and crystalline states along an axis. The IMC is implemented in the enhanced Monte Carlo (EMC), a self-developed code to investigate the nature of amorphous structure topological network-dependent mechanical properties [21,22,23]. The microstructure and mechanical properties relationships were successively studied by varying the topological entanglements such as brides, loops, and tails and strain rates. The semi-crystalline structure deformation under extension, compression, and shear were also determined using IMC and MD simulations [23].

Although noteworthy models have been projected to mimic HDPE structure and property predictions at atomistic and molecular levels, the associated complexities hinder the wide usage of IMC and induced crystallization strategies for plastic model preparation. The two most popular methods for preparing the semi-crystalline models, beginning from the most admired Rutledge et al. model preparation, need expertise, which is used to predict the microstructure characteristics at 350 K [17,21,22]. Similarly, the well-acknowledged Yamamoto’s model forms the crystals only when the model is being stretched at 350 K. In addition, other semi-crystalline models have been reported but still demand expert skill to prepare the most miniature lamellar stack model with minimal computational resources [24,25]. Generally, HDPE’s static and dynamic shear deformations processing temperatures are much higher than >400 K. The IMC and induced crystallization preparation methods evaluated the HDPE properties at 350 K, slightly higher than the desired experimental room temperature of 300 K, which might misinterpret the temperature-dependent processing condition or process optimization. Moreover, the mechanical behavior of plastics is sensitive to temperature and could cause the results to be miscounted. This limitation in the existing knowledge, and keeping in mind the temperature sensitivity in plastics processing, triggered us to prepare a minimalistic model in a size that can explain the plastic’s macroscopic information with reasonable accuracy, from which the non-experts can benefit. However, as the authors are aware, the suitable method appeared to be isothermal cooling from the melt at 300 K, which is the best strategy to prepare a stacked lamellar model since the evolving crystal growth is more realistic at 300 K. Indeed, the transferable potential for phase equilibria (TraPPE) force-field parameters must represent the interatomic potential, which has been well corroborated with the experimental data [18,26].

To ensure a systematic and replicable approach to preparing the most miniature HDPE model with lesser computational cost, Scheme S1 is recommended. A single chain of 1000 methylene units {(CH_2_)_1000_) = C_1000_} in its folded form is packed into four 2C_1000_ simulation boxes (two rectangular and two cubic) while satisfying the condition that at least the inter-entangled chains exist. The melting temperature of 450 K is adopted based on the processing temperature of HDPE in experiments [5]. After ensuring the satisfactory equilibration state, the lamellar stack-oriented model is prepared from isothermal crystallization of 2C_1000_ and extended to 10C_1000_, 15C_1000_, and 20C_1000_ chain models. We adopted a slow stepwise cooling strategy for our isotropic 450 K melt structure to gradually quench it to T = 300 K to avoid the unexpected glassy-state formation in plastics due to the fast-cooling protocols from the molten-state structure. Finally, the isothermal crystallization is performed to obtain the semi-crystalline structure form with a density of >0.90 g/cm^−3^ or until the time length of the simulations reaches 500 ns (0.5 µs). All the coil-quenched, and crystallized models are characterized by using the total density (ρ) and one-dimensional density profiles along the alternate lamella-oriented axis, degree of crystallinity (χ) from local order parameter {*P_2_*(r)}, and mechanical properties such as yield stress (σ_yield) and flexibility/elongation at break (ε_break). The characteristic metric demonstrates the formation of a lamellar-stack HDPE model. Using this approach, a reliable and computationally affordable model is anticipated to test the mechanical recycling or desired applications of plastic materials.

## 2. Simulation Methodology

### 2.1. Model Chain Preparation

#### 2.1.1. EMC Tool

**Figure 1:**
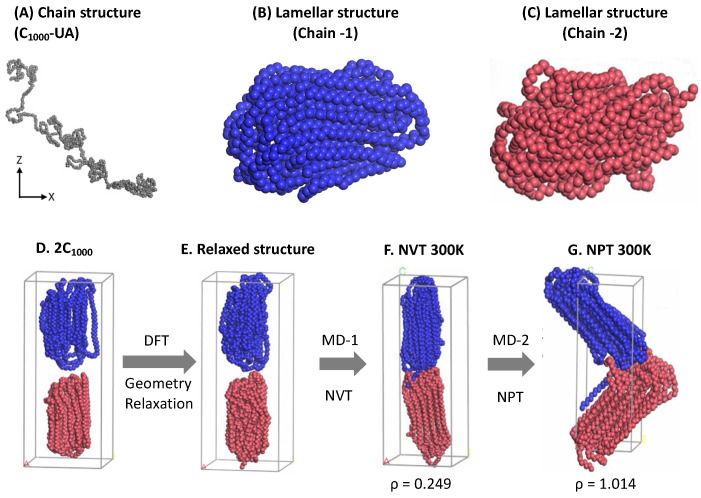
The pictorial representation of lamellar stacked model creation using the Material Studio 2022 (version 22.1.0.3462, Accelrys, San Diego, CA, USA): (**A**) the random chain created from the EMC tool; (**B**,**C**) shows the lamellae formation with folds; (**D**) two independent chains are kept in a stacked orientation; and (**E**) is the stacked structures relaxed form; (**F**) the NVT melting to 500 K and its NPT relaxation form is shown in (**G**) after 1 ns MD, respectively. Two independent HDPE chains’ lamellar stacked model (rectangular crystals) with folds and tails is used as an input data file in LAMMPS. The blue and brown colors represent independent C_1000_ HDPE chains.

EMC version 9.4.4. is used to prepare amorphous HDPE chains (ρ = 0.85 g/cm^−3^) comprising 500 monomer units at 300 K and 1 atm (Figure 1A) [27]. All the methylene (CH_2_) and the terminal CH_3_ units of HDPE chains are treated as UA, and each UA’s molecular weight is 14.01 amu. Initial C_1000_ is packed in a cubic box model dimension (a, b, c) of 30.2 Å. Molecular interatomic potentials are represented by the default Optimized Potential for Liquid Simulations (OPLS)-UA force field in the EMC tool.

#### 2.1.2. Material Studio’s Forcite Module

The EMC-created HDPE chains in the random and isolated forms are imported into the Material Studio visualizer to prepare thin, parallel layers or plate-like structures (the crystalline lamellar chain conformations), as shown in Figure 1B,C. Material Studio’s *Forcite* module lacks UA models’ force-field parameters, treating them as a single carbon atom with a molecular weight of 6.0 amu [28]. The interatomic potentials among all traces are represented by the universal force-field parameters (UFF) [29]. The electrostatic and Van der Waals interactions are summated using the group-based option available in the *Forcite* module with a cutoff distance of 12.5 Å. The calculations are initiated by heating the HDPE chains to 473 K (200 °C) (Figure 1A) using the Berendsen thermostat’s canonical ensemble (NVT) [30]. The simulation time for melting each single chain is 1 ns and a time step (Δ*t*) of 1 fs, and the medium energy calculation accuracy is maintained during the MD simulations. In the subsequent cooling phase, the NVT melted HDPE chain structure is coiled or globular and then cooled to room temperature (RT) at 300 K using the same 1 ns time length using similar MD simulations. The adopted simulation time lengths are sufficient to prepare the HDPE chains’ plate-like structures. Although the lamellar stems were obtained from the short 1 ns MD simulations, the lamellar structures in Figure 1B,C appeared to be satisfactorily folded lamellar chain structures. In the following step, i.e., Figure 1D, a sandwiched orientation of two lamellar chains is retained and relaxed by performing a complete geometry optimization using the innovative algorithm available in the *Forcite* module [28]. As mentioned, the relaxed structure is heated again with the NVT at 300 K and a 1 ns time length to properly bring the two chains to form, as displayed in the Figure 1F structure. Finally, the two lamellar stacked HDPE chains are further firmly packed in the rectangular cell box by allowing an isobaric–isochoric ensemble (NPT) MD simulation for a 1 ns time length and Δ*t* = 1 fs until the density reaches close to 1.0 g/cm^−^^3^. However, due to pressure constraints, the two chain conformations slightly penetrate each other and bend as the volume is compressed, showing the final form of 1.014 g/cm^−^^3^ at 300 K, as shown in Figure 1G. A single-point (SP) calculation is performed using the consistent valence force field (CVFF) force field [31] to convert them to a large-scale atomic/molecular massively parallel simulator (LAMMPS) input data file using the coordinate conversion *msi2lmp* tool available at the Sandia Laboratory [32,33]. This rectangular crystal (RC) model is used as input coordinate data as a starting structure in MD simulations by the LAMMPS code.

**Figure 2:**
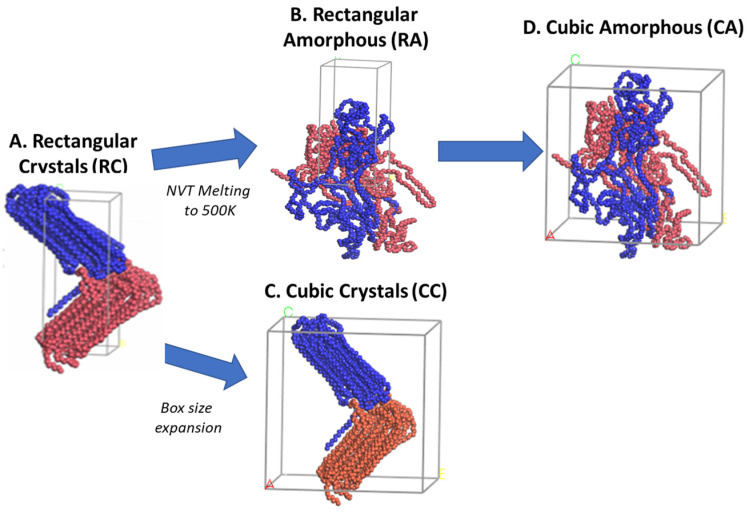
The creation of the other three models from the rectangular crystal (RC) form is illustrated. The RC model is melted at 500 K using NVT for 1 ns to obtain the RA model. At the same time, the cubic crystal (CC) and cubic amorphous (CA) models are created just by expanding the RC and RA box sizes in the x and y directions, respectively. The blue and brown colors represent each chain.

The RC model consists of packing two lamellar folded chain conformations in a sandwiched fashion (Figure 2A); however, to avoid the other potential possibility of giving the desired lamellar stacked orientation model with mixed chains conformation, the polymer chains in the amorphous state are not ignored, and hence, the rectangular amorphous model (RA) is prepared as Figure 2B by just melting the RC model to 500 K using NVT and the same parametrization as discussed in the previous paragraph. The 2C_1000_ initial models are expanded to cubic crystalline (CC) and cubic amorphous (CA) orientations alongside the prepared rectangular RC and RA models. The cubic box models introduce the CC and CA to the polymeric system representation due to their simplicity and suitability for the systems under consideration [34]. Figure 2C,D show the expansion of the rectangular box to a cubic box to prepare CC and CA, respectively.

**Figure 3:**
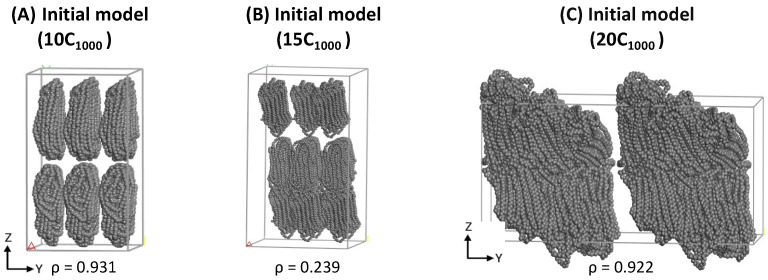
The LAMMPS input data files for extended 10-chain, 15-chain, and 20-chain models are using the 2C_1000_ RC model building Scheme S1 in Material Studio.

Simulated 2C_1000_ UA models are relatively tiny to convey their outcomes with the experiments, especially for semi-crystalline polymers. Additionally, the most significant aim of this study is to prepare an appropriate plastic model for mechanical recycling applications, so 10-chains (10C_1000_), 15-chains (15C_1000_), and 20-chains (20C_1000_) RC lamellar stack models can be based on the RC-2C_1000_ initial models’ ability to produce the alternate lamellar orientations in the simulations. Consistent with the 2C_1000_ RC model preparation, its extended 10C_1000_ and 15C_1000_ models are kept in the rectangular box along the *z*-axis lamellar stack, as shown in Figure 3A,B, respectively. However, the Y-directional stacked lamellar model is chosen for the 20C_1000_ model to check the capability of our strategy in preparing the semi-crystalline model with lamellar stack features in different dimensions of the cell box (Figure 3C). The LAMMPS data files for all the 2C_1000_ (RA, CC, and CA) and extended 10-, 15-, and 20-chain models are prepared employing the RC model preparation procedure. 

This study discusses the standardized force-field parameters (TraPPE) results obtained from using the LAMMPS code, and thus, deliberately, the discussion on the initially generated models from the Material Studio module is overlooked.

#### 2.1.3. Simulation Methods

The LAMMPS (version 7 August 2019) simulation parameters are measured using “real” units. The UA model description is the same as mentioned in the case of the EMC tool section. After conversion from the msi2lmp tool, each UA model’s re-assigned molecular weight is 14.01 amu. The potential energy functions representing the energy for the interatomic potentials among the atoms are described by TraPPE force fields. The choice of the TraPPE force field is based on its effectiveness in modeling HDPE crystallization and accuracy. Additionally, it can capture phase transitions such as crystallization and melting while excelling at representing the conformational behavior of polymer chains, including folding and packing. The simulations performed using the TraPPE force field will be close to the true nature of the HDPE [23]. In conclusion, the TraPPE force field is the best option to model and simulate the HDPE to prepare the stacked lamella-oriented microstructure with entangled brides, loops, and tails. Table 1 displays the mathematical expressions for the functional forms of the TraPPE force field and the details of potential parameters for HDPE models.

The primary MD simulation of the models begins from the structure relaxation by supplying the random velocities at 300 K, and the energy is minimized using a conjugate gradient algorithm in 5 × 10^5^ steps and Δ*t* = 1 fs in a microcanonical ensemble (NVE) at 300 K. Subsequently, the system rapidly heated from 300 K to 450 K for 1 ns using the canonical ensemble (NVT). It is immediately equilibrated isotropically at T = 450 K and P = 1 atm under isothermal and isobaric (NPT) conditions for a 10 ns time length. The steady states are confirmed by investigating the potential energy (PE), T, and total density (ρ) over the 10 ns equilibration, and the findings are discussed in detail in the Results and Discussion section. The default Nosé–Hoover thermostat and barostat are used in all simulations to control the temperature and pressure while adjusting the relaxation time after every 100-time step [35]. During the simulation, all the particles are binned 0.4 times the minimum distance between neighboring particles to improve the computational efficiency. To ensure the accuracy and reliability of the simulation results, the linear momentum of all atoms in three dimensions is equally used to stabilize the simulations. This prevents the artifacts that might occur due to the uncontrolled movement of particles. All NVT and NPT simulations were performed with Δ*t* = 2 fs and T, P, and V and ρ are regularly printed during the MD simulations. The bulk properties from MD simulations are assessed by imposing periodic boundary conditions (PBC) in all three directions. However, the spherical truncation (tail correction) is neglected beyond the cut-off distance (14.0 Å) [36].

#### 2.1.4. Isothermal Crystallization from Melt

The critical step of the MD simulations in this study is the isothermal crystallization of the equilibrated molten state model at 450 K. The well-equilibrated model is quenched and followed by isothermal cooling performed in three steps: firstly, the molten state gradually decreases from 450 K to 400 K with a 10 K/1 ns cooling rate in a stepwise manner, and the subsequent second step involves further temperature quenching of the model gradual cooling with a 5 K/1 ns cooling rate from 400 K to 300 K. In the final step, the system is isothermally cooled at 300 K for a maximum of up to 500 ns until ensuring the formation of the lamellar stems and recording the total density evolution from amorphous ~0.85 at 450 K to the normal crystalline HDPE density range of 0.90–0.94 g/cm^−3^ at 300 K. The T quenching and isothermal cooling trajectories are recorded every 10 ps and 1 ns time interval to analyze the crystal kinetics and structural transformations.

### 2.2. Models’ Characterization

#### 2.2.1. Density and One-Dimensional Density Profile

All the prepared models are characterized by a ρ-profile diagram along all axes’ directions concerning the simulated box. The density is computed using the chunk option available in the LAMMPS by partitioning the box for 0.5 Å bin. The density could then be the number of atoms in the bin divided by the bin size or volume. A one-dimensional density profile is computed in all 2C_1000_ models to rationalize the physical states among the microstructures quantitatively. These resemble density profile diagrams that justify the semi-crystalline HDPE model with the lamellar-stack orientation [20,21,22,23,24]. The quantitative justification of the alternate lamellar stack model formation is for all 2C_1000_ models using NVT-ensembled MD simulations of 10 ps with 0.1 ps trajectory samples collected in the density profile calculation of the semi-crystalline model in the Z-direction at 300 K.

#### 2.2.2. Degree of Crystallinity (χ) from Local Order Parameter P_2_(r)

The local crystalline order is a crucial parameter in understanding and tailoring polymeric material, and it also controls the mechanical behavior of polymers [12,37]. The extent of crystallization is traced from the local-order parameter [*P_2_*(r)] calculation with the aid of the coarse-grained molecular dynamics program (COGNAC) in the open computational tool for advanced materials technology (OCTA) [38].

The simulation cell is initially divided into the small 8 Å mesh-sized regions in the x, y, and z directions. To describe the explicit position of the atoms within a polymer chain, the chord vector ($b_{i}$) is defined as the geometric midpoint between two consecutive atoms along a polymer chain and determined as the average of the positions of these two atoms. Then, the chord vector coordinates are assigned to their 8 Å respective mesh cells. The local order parameter $P_{2}\left(r\right)$ is calculated using the following equation from the defined mesh cells.

$$P_{2}\left(r\right)=\langle 3{cos}^{2}\theta_{i,j}-1\rangle /2$$

The angle ($\theta_{i,j}$) between chord vectors *b_i_* and *b_j_* within the same mesh-cell size of 8 Å is located at the representative position $r_{i}$. The average is taken over all pairs of chord vectors within the mesh cell.

The degree of crystallinity (χ) is computed from the number of mesh-cells ($N_{c}$) with local order parameters as recommended by Yi et al., threshold 0.4 (*P_2_(r)* > 0.4) divided by the total number of mesh-cells in the system (*N_total_*) [39,40].

$$\chi =N_{c}(P_{2}>0.4)/N_{total}$$

All isothermally crystallized models χ are computed at every 50 ns time length interval evolution during the isothermal crystallization at 300 K to judge the formation of the alternate lamellar orientation along an axis.

#### 2.2.3. Entanglements Analysis

The number of entanglements per chain <*Z>* in all models is estimated from the *Z1+* code developed by Kröger et al. [41]. The key average number of entanglements per chain parameter <*Z>* is calculated for all models in their equilibration state at 450 K, quenched model state at 300 K, and isothermally crystallized model again at 300 K. All three chosen physical states represent their characteristic microstructure, and having their topological information gives the clear mandate of chain packing in temperature-dependent phase transformation of simulated models.

#### 2.2.4. Mechanical Properties

The intrinsic properties of the polymeric materials are their uniaxial tensile strength uploading with the strain (ε) at specific temperatures [16]. Moreover, mechanical properties are characteristic features of plastic materials and illustrate the mechanism involved with the internal structure, i.e., microstructure arrangement. Since the semi-crystalline structure consists of both amorphous and crystalline states, its mechanical properties are unique and are a characteristic metric for validating the models and experimental data at 300 K. It is the best metric to validate the model’s reliability while delineating material characteristics’ stiffness, stability, and ductileness at a molecular level. The extended 10-chain, 15-chain, and 20-chain models are uniaxially deformed with the strain rate ($\dot{\varepsilon }$) of 10^10^ s^−1^, using the NPT conditions at T-300 K and zero-pressure for the two lateral simulation cell faces, which are well studied for HDPE-UA models by Hossain et al. [42]. The uniaxial deformation is performed in the lamellar stack-oriented *z*-axis direction for 10- and 15-chain models. On the other hand, it is in the *y*-axis directional deformation for the 20-chain model. The stress components (p_xx_, p_yy_, and p_zz_) are obtained from the Virial theorem, which sums up the forces between particles and their positions.

The stress–strain (S-S) curve is obtained by converting the pressure tensor in the atmosphere to megapascals (MPa), and the strain is represented in the percentage (%) of elongation of the simulation box in one axis direction. The ε is computed using the following formula:

$$\varepsilon =(\left(L-{\;L}_{0}\right)/L_{0})=(\Delta L/L_{0})$$

where ε is the uniaxial deformation strain; *L* is the final length of the box; and $L_{0}$ is the initial length of the box. Whereas the $\dot{\varepsilon }$ is fixed to be 10^10^ s^−1^ and mathematically represented using the following formula:

$$\dot{\varepsilon }=(\Delta \varepsilon /\Delta t)$$

where $\dot{\varepsilon }$ is the uniaxial deformation strain rate, Δε is the change in strain, and Δt is the change in time. All the $\dot{\varepsilon }$ values used for deformation studies agree with the literature reports on HDPE models when the stiffness and ductility are compared [21,23,42]. All the simulation boxes are elongated in uniaxial deformation in the alternate lamellar-orientated direction, and the elongation is performed for a 1 ns time length and Δ*t* = 1 fs [17,37,43]. The $\dot{\varepsilon }$, however, is much faster than experiments. Nevertheless, a marginal attempt is made to reduce the loading with Δ*t* = 1 fs. To observe the evolution of the microstructure during the uniaxial tensile deformation simulations, the coordinate trajectory is scrutinized at regular intervals of 1 ps.

Three initial samples of the same structures are considered. Their average properties are characterized by a one-dimensional density profile along an axis, number of entanglements, and SS curve from uniaxial strain deformations. All the graphical visualizations are shown using the OVITO tool version 3.9.4 [44], except for the degree of crystallinity, which is exclusively obtained from the OCTA tool version 8.4.

## 3. Results and Discussion

Individual single-chains-packed rectangular and cubic box 2C_1000_ models’ simulations results are taken as a cornerstone for building the more reliable moderate number of chains (10C_1000_, 15C_1000_, and 20C_1000_) models, which are intended to simulate the actual macroscopic behavior of plastics. Once the packing and crystallization of all 2C_1000_ models are completed, the obtained models are characterized by computing the model’s total densities (ρ), density along the one-dimensional axis, and degree of crystallinity (χ). A 2C_1000_ model gives alternative lamellar orientation, irrespective of the nature of the simulation box among RC, RA, CC, and CA; the larger-sized models with 10, 15, and 20 HDPE chains are prepared similarly. Additionally, unraveling the model’s capability in deforming under external stress is measured by applying uniaxial deformation strain and extracting the stress–strain curve (SSC) information from ~500% deformation of 10C_1000_, 15C_1000_, and 20C_1000_ models. The advantages and limitations of our models are discussed in the Conclusion section.

### 3.1. Model Structure

#### 3.1.1. Equilibration of 2C_1000_ Models

**Figure 4:**
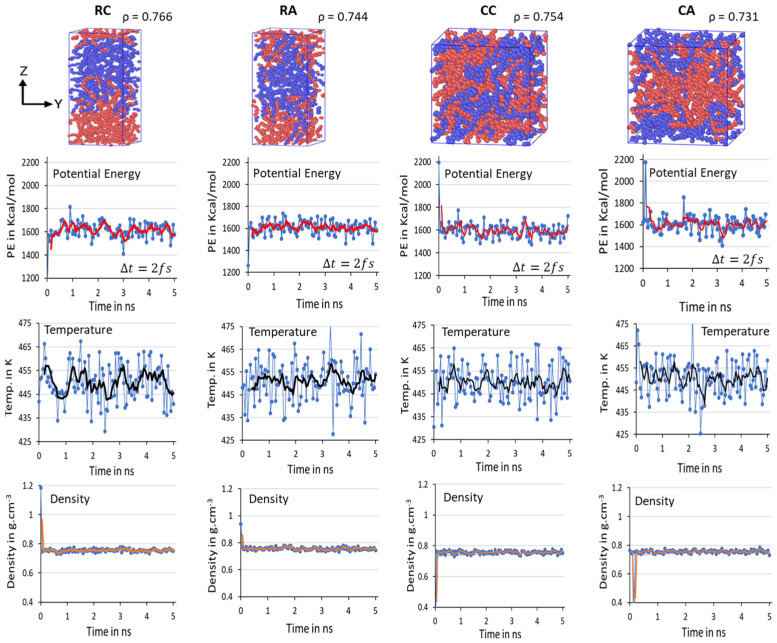
All equilibrated RC, RA, CC, and CA models and their densities (*ρ*) are shown as equilibrated structures at 450 K and 1 atm for 10 ns time length. Each color represents a C_1000_ chain. Mean average values of potential energy (PE) in red lines, temperature (T) in black lines, and density (ρ) in orange lines are computed for the equilibration of 2C_1000_ rectangular and cubic box models. The blue dots in respective figures represent the actual potential energy, temperature, and densities at respective simulation times.

Initial 2C_1000_ models with lamellar sandwiched and random mixed chain conformations are packed in rectangular (RA and RC) and cubic (CA and CC) cell boxes and are equilibrated, as shown in Figure 4. Due to the discrepancy in the chain conformation and orientations of the chains, it is essential to confirm the model’s steady state equilibration before progressing to the crystallization by quenching followed by cooling at 300 K. Consequently, the equilibration parameters such as PE, T, and ρ of all models over time are attested for all four 2C_1000_ models in Figure 4.

It is clearly illustrated from the above figure that the mean average values of PE, T, and ρ reported with the evolution of a 10 ns time length to validate the deviation of the parameters from the desired results are stabilized over time. The balanced PE, T, and ρ in NPT equilibration for 10 ns dictates the steady state obtained concerning the T at 450 K and ρ ca. 0.770 g/mol*^−^*^3^, in addition to the steady PE within a two- ns time length (in Figure 4, it is labeled as 1 to 5 ns, respectively, with Δ*t* = 2 fs). No significant changes appeared when the MD simulations are further prolonged to 10 ns. The slight discrepancy in the averages of equilibration parameters is under the well-accepted (~5%) range from the desired values, indicating the reliable computational protocol practiced in this study, and ensuring the formation of the crystallized models might be susceptible to predicting plastic’s deformation applications. The results demonstrate that the well-equilibration state is reached in all models, and the 10 ns NPT equilibration is sufficient. Though the steady state is achieved in all four models within a 2 ns time length, the two chains might not be completely packed in the RC model, which might question the stability of the crystallized model. The 2C_1000_ model initially attempts to make the chains packed in a rectangular cell and crystallized to form an alternate lamellar orientation along an axis. Consequently, an extensional study involving a greater number of chains must be included. Since the model size is relatively tiny to obtain reliable plastic macroscopic properties, examining a more significant number of HDPE chains is recommended and carried out and is discussed later.

#### 3.1.2. Crystallization of 2C_1000_ Models:

**Figure 5:**
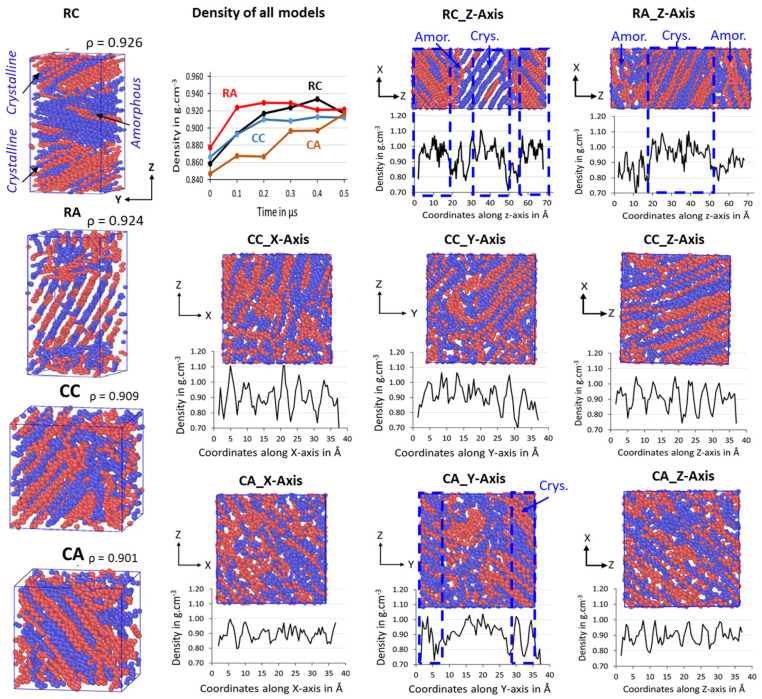
The total density evolution over time in the isothermal cooling at 300 K of RC, RA, CC, and CA models for 1 μs. A one-dimensional density profile is computed in the *Z*-axis direction for RC and RA and all-axis densities for CC and CA. The ordered and random regions in the box represent the crystalline and amorphous states of the HDPE, respectively. The blue and brown colors represent each chain.

The semi-crystalline microstructures of the RC, RA, CC, and CA models are shown in Figure 5, along with their ρ in g/cm^−3^. The 500 ns isothermal cooling results show that the four 2C_1000_ packed models successfully formed the ordered lamellar crystals and the random amorphous states. Irrespective of the dimension of the simulation box, all four models packed in both rectangular and cubic models are semi-crystalline at T = 300 K and P = 1 atm, and their respective densities between the 0.901 and 0.926 g/cm^−3^ range, indicating the formation of actual HDPE semi-crystalline structures. Since the interacting pieces are just UA models, the crystallized regions are exclusively non-covalently interacting by weak van der Waals (vdW) forces. All the model’s crystallizations are carried out for 500 ns; the cubic models, i.e., CC and CA, show a lower crystallinity with 0.909 and 0.901 g/cm^−3^ than that of the rectangular box models, RC with 0.926 g/cm^−3^ and RA with 0.924 g/cm^−3^. Whereas the crystallized 2C_1000_ models RC, RA, CC, and CA packed chains are obtained in divergent crystalline orientations, nevertheless, Figure 5 also shows the formation of alternate lamellar orientations in the RC model (Figure S1), which shows the microstructure lamellar and amorphous configuration alternately, directing the way to obtain a larger model to assess the properties of the macroscopic plastic using multiscale modeling.

The chain mixing in the RC-2C_1000_ model might need to be improved. However, the primary aim of this study is to obtain a stacked lamellar orientation. The RC model exhibits an auspicious lamellar stacked conformation with desired microstructure features further confirmed by density and crystallinity data performed on the well-equilibrated system, as shown in Figure S2. Generally, HDPE semi-crystalline materials are illustrated at 300 K by their respective crystallinity between 0.940 and 0.970 g/cm^−3^ [45]. The density of the isothermally cooled RC model is 0.941 g/cm^−3^, which is in the desired range for the HDPE semi-crystalline structure. It further implies that the densities represent the highly ordered PE chains with sufficient random or zigzag amorphous regions that could be entangled. Figure 5 shows the density evolution over time for all 2C_1000_ models, and it has to be noted that the crystallization is achieved for all models within 300 ns of time length with ρ close to 0.90 g/cm^−3^. Relatively, rectangular box models crystallize faster than their counterpart cubic models, and the ordered density in the models follows RA > RC > CC > CA. Results imply that the studied models have the potential to form ordered lamellar stems with a few 100 ns time length MD simulations, and the chains packed box has not influenced the crystallization characteristics, except for the starting structure orientation.

**Figure 6:**
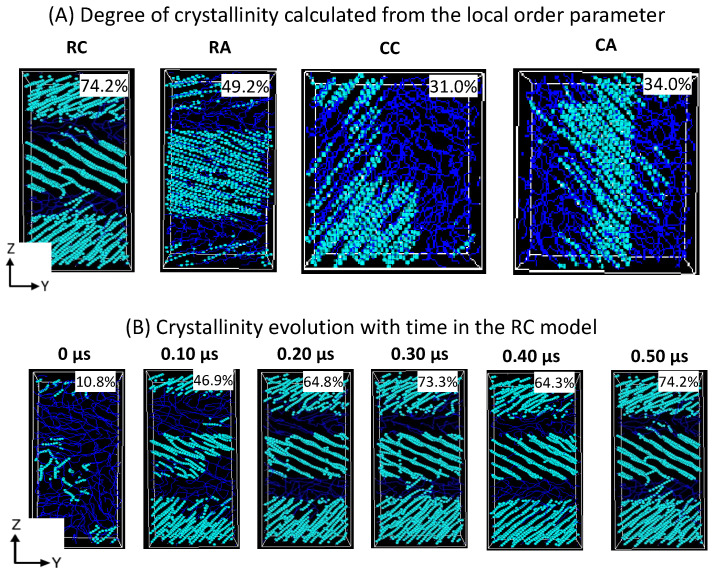
Schematic representation of the crystalline and amorphous regions of (**A**) 2C_1000_ (RC, RA, CC, and CA) models and RC model’s degree of crystallinity evolution in the isothermal crystallization at 300 K. (**B**) The formation of three-layer lamellar stems is being illustrated for the RC model in isothermal cooling at 300 K. The cyan and blue colors represent the crystalline and amorphous regions, respectively.

The formation of a desired alternative lamellar structure within the box, a one-dimensional density profile, is implemented on all 2C_1000_ models, along the *z*-axis in rectangular boxes, and x, y, and z-axes in all cubic models. Figure 5 shows that the lamellar-stack orientation is obtained in only RC models. In contrast, such orientations have not appeared in any other models, indicating that the RC model’s initial structure supports the formation of the alternate lamellar orientation even though the two chains’ equilibration has yet to appear to be mixed appropriately. It could be assumed that the more significant number of chain models prepared with such a strategy would build the anticipated model with the compatible chain mixing, and investigations in that direction should not be ignored. To assess further, the degree of crystallinity for all 2C_1000_ models is reported in Figure 6A, and the same is listed for the RC model in its isothermal crystallization at 300 K for a 500 ns time length using the local order parameter calculations. The degree of crystallinity of RC from 0 ns to 500 ns with a 100 ns time interval is reported in Figure 6B. The model is confirmed to be highly crystalline, with 74% crystallinity. The lamellar texture alternates along the *z*-axis. Such an orientation order has not appeared in the remaining models, supporting the RC model’s suitability in preparing the lamellar stacked HDPE models. 

The reason for choosing the rectangular box is its suitability for simulating anisotropic systems, where the physical properties differ along the axes, and its accuracy in mimicking the actual conditions [34,46]. Undeniably, the choice of the extended models is claimed based on the results obtained for the RC-2C_1000_ model characterization. A rectangular MD simulation box would offer flexibility and could replicate the anticipated mechanical recycling conditions adopted in the rheometric experiments, where the mechanical recycling approaches involving non-equilibrium MD (NEMD), such as static and dynamic shear deformations of the simulation boxes to a more significant extent, for example, large amplitude oscillatory shear (LAOS) deformation will be simulated [17,47]. In addition, the critical aspect of this MD simulation is obtaining an alternate lamellar structure for the HDPE to explore a microstructure–mechanical property relationship and highlight the molecular deformation mechanism in different conditions realized in mechanical recycling methods. The chains in the RC need to be mixed better in the equilibration. Nevertheless, the model has the potential to form a stacked lamellar orientation in the MD simulations. So, its preparation strategy is chosen for extended models.

### 3.2. Extended Models (10C_1000_, 15C_1000_, and 20C_1000_)

#### Equilibration of the Extended Models

**Figure 7:**
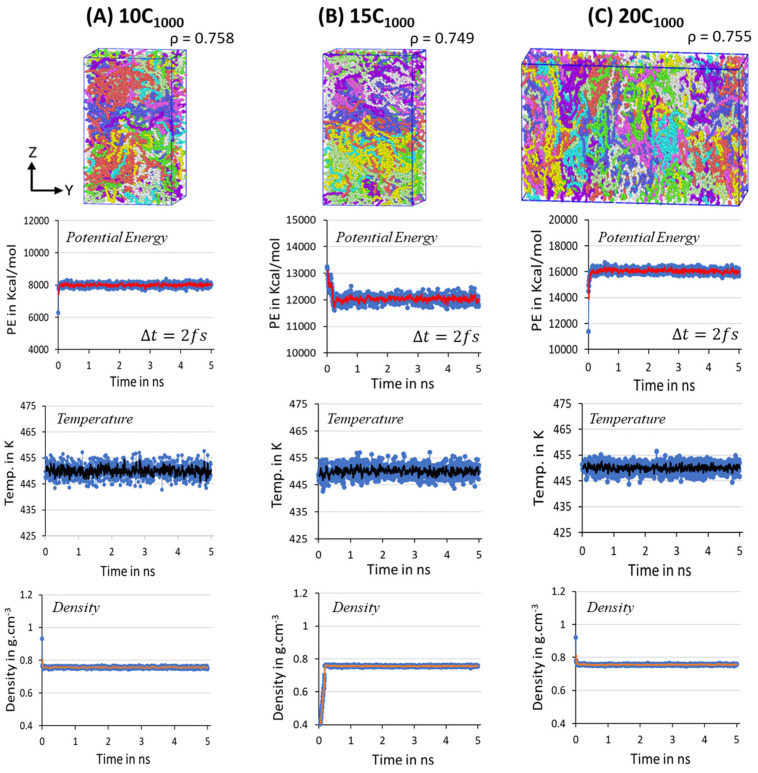
The potential energy, temperature, and density parameters stabilization in the NPT equilibration for 10 ns is shown for 10-, 15-, and 20-chain models at 450 K and 1 atm. The blue dots in all the figures are the potential energy, temperature, and densities obtained during the MD simulations. Whereas the lines with red, black and orange are the moving averages of the same, respectively. Each color in the figures represents an independent chain in the model.

The structural segments of amorphous and crystalline regions and the density close to experiments are achieved within 500 ns MD simulations for a minimalistic HDPE 2C_1000_ model. Likewise, in 2C_1000_ simulation models, a similar MD simulations equilibration strategy has also been applied to the 10-, 15-, and 20-chain models. All the extended models possess the initial lamellar conformations within the rectangular boxes shown in Figure 3. Despite the initial models with rectangular box and crystalline chain conformations, the equilibration parameters PE, T, and ρ quickly reached the stable range in all three studied models. In the extension of the simulations over twons to 10 ns (in the figure, it is labeled as 1 and 5 ns, respectively, with Δ*t* = 2 fs), the presence of the steep moving average lines in Figure 7 suggests that the system is sufficiently equilibrated, and no further MD run is needed. Unlike the 2C_1000_ models, the fluctuations in the larger models are reduced, and it is <5% from the desired MD conditions, indicating that as the size of the box increased, the noise fluctuation in the equilibration parameter is reduced, as seen in the Figure 7A–C.

**Figure 8:**
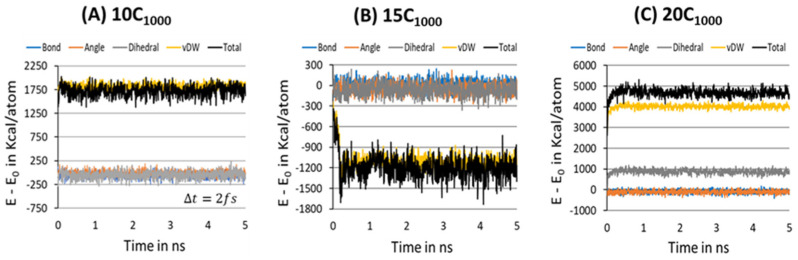
Potential energy decomposition into the various components for 10-, 15-, and 20-chain models at 450 K for the equilibration step to ensure the stabilization of the microstructure.

To further ensure the formation of a stable microstructure system in the equilibration step, the energy decomposition of HDPE model systems is gauged for a MD simulation time scale length of 10 ns, and each snapshot is recorded after each 200 ps, where the Δt=2 fs. Figure 8A–C represents the energy decomposition components such as bond stretching, angle bending, dihedral torsion, and van der Waals forces among the PE chains; it is evident that all are stabilized in within a 2 ns time length scale. On the other hand, the total energy appears to be distinct for the 15C_1000_ model and 10/20C_1000_ models, which could be attributed to the lamellar stem arrangements in the starting structure. Figure 3A–C illustrates that the lamellar layers are vertically arranged rather than horizontally, as in the case of the 15C_1000_. The energy components in the 10- and 20-chain models are similar because the 20C_1000_ model’s initial structure is prepared by combining two 10C_1000_ models. Despite the preparation of the initial models being different, the equilibration is sufficiently reached. A well-equilibrated HDPE model is anticipated before the production run for isothermal cooling to obtain a semi-crystalline model.

**Figure 9:**
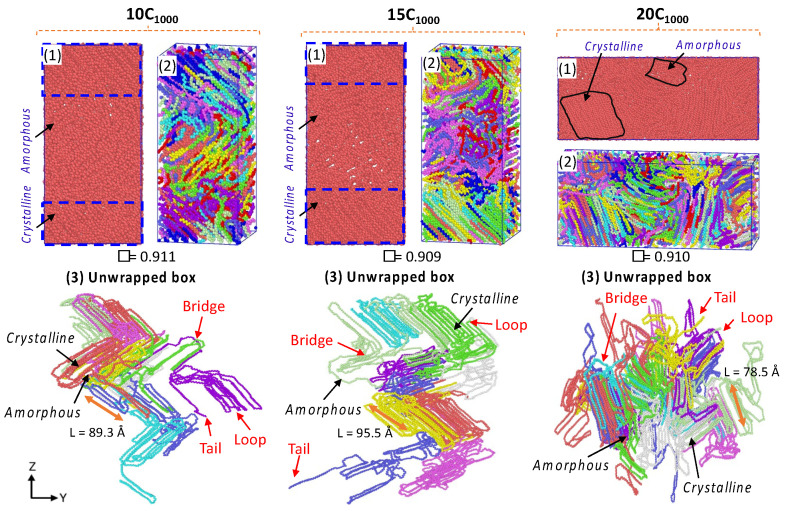
Semi-crystalline extended models: 10C_1000_, 15C_1000_, and 20C_1000_. The unwrapped box/open box models indicate the formation of the crystal network’s bridge, loop, and tails. Each color in the figures represents an independent chain in the model.

For the temperature quenching process, the penultimate step for the isothermal cooling at 300 K, the density transformations in all extended models from the 450 K melt structure to 300 K cooled structures are shown in Figure S3. It is concluded that the temperature quenching process gradually increases the density from ~0.760 g/cm^−3^ to the desired ~0.850 g/cm^−3^ at 300 K, acknowledging the reliable Trappe force-field parameters chosen for modeling. The main extended (10C_1000_, 15C_1000_, and 20C_1000_) semi-crystalline models obtained in the MD simulations after the 500 ns isothermal cooling at T = 300 K and P = 1 atm are shown in Figure 9**.** The microstructures with ordered crystalline lamellar (in blue dotted lines) and random amorphous segments having bridge, loop, and tail crystal networks, which can be seen as a part of the entanglement regions, and [40,48,49] are consistent with previously reported computational models [50]. The MD simulation models of 10, 15, and 20 chains show a more realistic HDPE nature than the RC-2C_1000_ model. This is anticipated because many chains facilitate chain compatibility, and their microstructure is crucial in understanding the macroscopic polymer material behavior. Figure 9 also illustrates the microstructure in the unwrapped box form, a more meaningful physical form of the crystal network entangled with several PE chain structural segments, as described in detail by Rutledge et al. and Yamamoto et al. [12,17,48]. The concluded data suggest that the possibility to model a more justifiable semi-crystalline HDPE model using a moderate number of chains is possible using MD simulations, which gives characteristic microstructural features and helps in correlating the structure–property relationship.

The obtained densities for all models are within the range of 0.90 to 0.911 g/cm^−3^, a marginally underestimated value than the experimental value of 0.94 g/cm^−3^ in Table S1, and the smaller crystal stem length range from 78 to 95 Å of the unwrapped model forms is also evident in Figure 9. The lamellar stem orientations are parallel and are in the z-direction of the box axis. In contrast, the orientation in the 20C_1000_ model is in the *Y*-axis direction. The lamellar stems are heterogeneously formed in the simulations. Though the obtained densities are lower, the lamellae texture appear to be highly oriented crystal chains. The box dimensions from Table S1 also show that the quenching and isothermal crystallization shrink the box dimensions as the density increases, suggesting the ordering of the lamellae due to the contraction of the simulation box. This phenomenon represents the spontaneous formation of the crystals during the MD simulations. It is also an accurate mimic of the experimental crystallization process, where the sequential formation of the crystals appears in the temperature quenching and isothermal cooling conditions.

For reference, each chain behavior in isothermal crystallization at 300 K and 1 atm for the 10C_1000_ model is analyzed, which involves the folding of the chains with progression of time, which further converts it to the stiffer lamellar structure with extended MD simulations to 500 ns; all the graphical illustrations are shown with 50–100 ns time intervals in Figure S4. Consistent with our assumption, the crystallization is achieved in each chain within a 100 ns time length in the 10C_1000_ model. However, the extended simulation models have reached saturation at a 250 ns time length and have not progressed significantly, even with prolonged simulation extension to 500 ns. The isothermal crystallization of chains summarizes that the extended models could form the stacked lamellar orientation within a few 100 ns MD simulations. It supports this strategy as promising to prepare such models for plastic materials applications.

### 3.3. Characterization of Extended Models

#### 3.3.1. Density in Isothermal Cooling at 300 K

**Figure 10:**
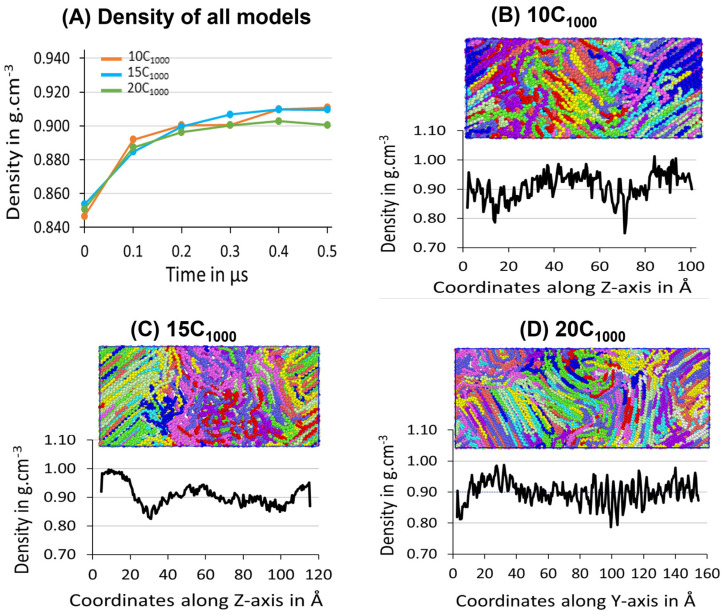
(**A**) Density evolution for 10-, 15-, and 20-chain models is shown during the isothermal crystallization at 300 K and 1 atm. Additionally, the Z-directional densities in the case of 10 chains (**B**) and 15 chains (**C**) and Y-directional in 20-chain models (**D**) are shown. The ordered and random regions in the box represent the crystalline and amorphous states of the HDPE, respectively. Each chain represents an individual chain in the model.

The three samples’ average density during the 500 ns isothermal crystallization for each extended model is presented in Figure 10A, which illustrates the density convergence near 300 ns, which does not change when the simulation is further extended to 500 ns, which indicates that the crystallization for the extended models reaches the saturation level. The obtained semi-crystalline structures are suitable for deformation applications. This confirms that the minimum stable model to be considered from this strategy is 10C_1000_ HDPE. However, crystal growth occurs rapidly from the beginning of the isothermal crystallization process, supporting the melting and sequential quenching at 450 K, followed by isothermal cooling at 300 K, which could be an ideal condition for HDPE crystallization at 300 K-T and 1atm-P within 500 ns using TraPPE interatomic force-field parameters, which is proven to give a much faster crystalline HDPE model.

The experimental amorphous state density is 0.85 g/cm^−3^, similar to the values obtained for the models 10C_1000_, 15C_1000_, and 20C_1000_ after quenching at 300 K (Figure 7). The mass densities evolution over time for the 10C_1000_, 15C_1000_, and 20C_1000_ final structures are in the range of 0.90–0.911 g/cm^−3^, which is slightly lower than the experimental values of 0.94–0.97 g/cm^−3^. Though the obtained density data are slightly lower in the crystalline forms, the experimental values of well-defined crystal lattices and amorphous regions are formed with sufficiently longer lamellar lengths (Figure 9).

#### 3.3.2. Determination of Lamella-Stack Orientation

The semi-crystalline lamellae orientation has interestingly alternative crystalline lamellae stems and random amorphous segments with tails, bridges, and loops, which makes this model a sandwich structure. Such models are examined for the full atomistic and UA model’s microstructure–mechanical property relationships in HDPE [50]. The final snapshot of the simulated models from isothermal crystallization (Figure 10) shows the density profile along the microstructure with alternating crystalline and amorphous regions in the *z*-axis for the 10-chain and 15-chain models and *Y*-axis for the 20C_1000_ model. The lamella c-axis direction is seen in the Z-direction for the 2C_1000_, 10C_1000_, and 15C_1000_ chain models, while it is in the Y-direction for the 20C_1000_. Therefore, the density is computed along the C-axis direction to rationalize the lamellar-stack orientation. Among all the models, either a two- or a three-layer alternative lamella orientation appears. Still, exclusively, the 10- and 15-chain models exhibit a clear lamellar-stack orientation along the *z*-axis, while the 20-chain model’s *Y*-axis shows similar density variation along the axis [19].

The densities along the lamella C-axis represent the concrete physical nature of the amorphous and crystalline segments, and the amorphous region possesses a ~0.85 g/cm^−3^ density, while the crystalline region possesses the same with ca. 1.0 g/cm^−3^. Nevertheless, the lamellar stem sizes vary; as the number of chains increases due to decreased density, the lamellar stem length decreases, as shown in Figure 9. The 15C_1000_ microstructure density represents a stiffer lamellar stem; the density is ca. 0.95–1.0 g cm^−3^. In contrast, the densities along the Z-direction in the 2C_1000_ model fluctuate because of the smaller size of the model, which indicates the unrealistic nature of the material, which is overcome by increasing the model size to 10C_1000_, 15C_1000_, and 20C_1000_. The density distribution in the simulation box is attributed to the alternating lamellar and amorphous states along one dimension; such models would help to predict the structure–property relationship in plastics to optimize the processing condition with an enhanced mechanical property of the recycled HDPE pellets. A systematic shear deformation work in this direction is being considered.

#### 3.3.3. Degree of Crystallinity (χ) in Extended Models

**Figure 11:**
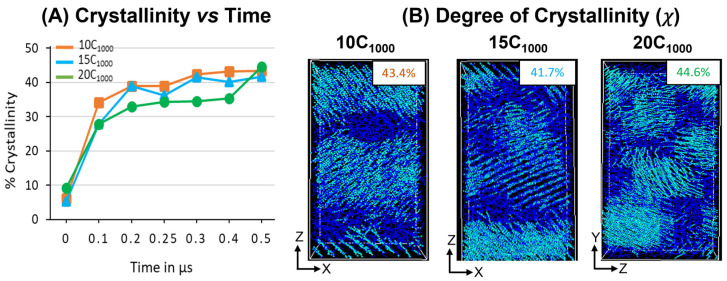
(**A**) The degree of crystallinity evolution in the isothermal cooling at 300 K for 10-chain, 15-chain, and 20-chain models. (**B**) Representation of crystalline (cyan color) and amorphous regions (blue color) of the final trajectory file (500 ns).

The degree of crystallinity (χ) is analyzed in extended models over 300 K, as shown in Figure 11A. A steeper jump in the χ values for all models between 0 ns and 100 ns indicates that the temperature quenching followed by isothermal cooling of the isotropically equilibrated model can yield a faster crystallization (<100 ns time length of MD simulations from the melt) and corroborated with previous reports highlighting the computational protocols that are adopted to prepare the semi-crystalline models using MD simulations [16,17]. The χ values trend of 10 chains > 15 chains > 20 chains illustrate that with an increase in the number of HDPE chains, the crystallization is slower, which can be attributed to the fact that the bulk size of the chains hinders their movement with an increase in the number of topological entanglements. However, from the 400 ns time length, the χ values trend changes to 20 chains > 10 chains > 15 chains due to a sudden jump in the χ values for the 20-chain model, which might be due to the significant reordering of the molecular chains to become ordered crystalline chains during the MD simulations time from 400 ns to 500 ns. Although with the variation in the number of chains and small-size models, when the macroscopic behavior mimics a material, such as HDPE plastics, it mostly stayed the same, supporting that the 10C_1000_ model could be used to study the appropriated plastic recycling strategies.

Similarly, a 500 ns snapshot microstructure trajectory file comprising crystal (cyan color) and amorphous (blue) regions in Figure 10B is segregated to verify the formation of alternate lamellar orientations in the simulations box along an axis. It is observed that the semi-crystalline HDPE models with crystalline and amorphous regions are prepared with χ ~42–45%, and alternative crystal lamellar and amorphous regions are depicted using the local order parameter calculations for 10- and 15-chain packed models. On the other hand, slightly directionless stacked lamellar orientation with chains increased from 15 to 20 and appeared heterogeneous. Although the lamellar texture distributions are dissimilar, the high order of crystalline regions in alternate ordering dominates all models, which is anticipated in our MD simulations. The obtained models are semi-crystalline, and their microstructure has an alternate lamellar orientation that aligns with the reported literature and could be used for microstructure–property relationships [16].

The chain ordering behavior from the beginning of the isothermal crystallization for all models is also shown in Figure S5. The χ for 10C_1000_, 15C_1000_, and 20C_1000_ models from the initial MD step 0 ns to the final 500 ns (0.5 µs) time step evidenced that the crystal growth is multi nuclei that grow with fast chain ordering in the prolonged cooling. The driving force for ordering the chain could be attributed to the vdW forces among the chains. As the initial stage of the model shows the ordering of the chains, it should be noted that the quenching of the chain itself has the seeds for the crystal nuclei. During the MD simulations, the isothermal crystallization temperature is critical for crystal growth and chain ordering to form lamellar stems.

#### 3.3.4. Entanglements Analysis Using Z1+ Code

We quantitatively assessed the entangled polymer chains at different stages of the simulation conditions. Table 2 lists the directly measured mean number of kinks per chains (<*Z*>) for all the models using the *Z1*+ code developed by Kröger et al. [41]. The listed data registered with the equilibrated model at 450 K, followed by the quenched model (from 450 K) at 300 K, and the critical isothermally crystallized model at 300 K in all 10C_1000_, 15C_1000_, and 20C_1000_ models. The consistent trend across all models indicates that at 450 K, the lower entanglements in the coiled structure due to thermal expansion of the polymer chains lead to more extended and less kinked conformations. As the temperature quenched to 300 K, the number of kinks per chain increased, indicating the improved polymer chains’ compactness even in the amorphous state. Finally, the kinks per chain decreased after isothermal crystallization due to the partial ordering of the chains, leading to moderate kinking, a true nature of a semi-crystalline structure [16].

Fortunately, the entanglement density reported for linear polymers at 443 K is 12.2, marginally a little higher than the computed data, showing that the characteristic signature of an entangled plastic model is achieved during the MD simulations corroborated with temperature-dependent physical states of entangled polymers [43].

#### 3.3.5. Mechanical Properties

It is evident that when uniaxial deformation is applied in the lamellar-stack orientation, a unique mechanical behavior of HDPE semi-crystalline materials justifies the formation of lamellar-stack orientation in the obtained model [17,37]. Various studies, including X-ray scattering experiments (SAXS and WAXS) and a few computational studies, have shed light on the HDPE material’s SS curves comprising a sharp/linear elastic region peak followed by the high yield point and plasticity. Finally, the breakpoint/ultimate plastic deformation due to cavitation in the amorphous region is a common phenomenon of their molecular mechanism. It is termed as the cavitation mechanism [21,22,50,51,52]. Figure 11 illustrates authentic SS features for 10C_1000_, 15C_1000_, and 20C_1000_ models that mimic the HDPE characteristic SS curves. On the other hand, the SS curves for 2C_1000_ models are ignored due to the imposed boundary conditions and small size, which might underestimate the results. The uniaxial deformation or tensile test is performed for the 2C_1000_, 10C_1000_, and 15C_1000_ models along the stacked lamellar orientation in the Z-direction, for the 20C_1000_ model, it is deformed in the *Y*-axis direction. For plotting the SS curves, we considered the moving average of the pressure tensors for all extended models.

**Figure 12:**
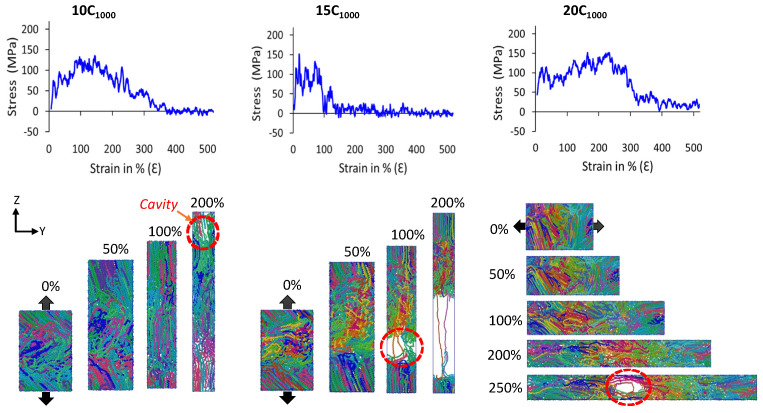
The uniaxial deformation (tensile test; stress–strain curve) to a strain rate of 10^10^ s^−1^ is performed at 300 K and 1 atm pressure for the extended models. Models 10C_1000_ and 15C_1000_ are deformed in the Z-direction, while Y-direction deformation is performed in the case of the 20C_1000_ model. All the deformations are performed along the alternate lamellar orientation axis. As the trajectory frequency is recorded at 1 ps time intervals, the mean average lines, in some cases, starting stress points are not visible. However, the starting stresses exist at low-frequency trajectory recordings (0.1 ps). Each color represents an independent polymer chain in the simulation box.

The mechanical testing of models in all axes shows anisotropic SS curves. Further keen observation of SS curves indicates that the *Z*-axis in the 10C_1000_ and 15C_1000_ models is alternate, whereas it is alternate for 20C_1000_ along the *Y*-axis. Figure 12 illustrates that the lamellar textures are alternate in only one direction, and the remaining axis in the models, especially in 20C_1000_, are not perfectly stacked in one direction, which is attributed to the fact that their mechanical behavior is anisotropic and further clarifies that the microstructure is heterogeneously distributed [17,20]. However, the mechanical properties are anisotropic due to the internal lamellar stems being oriented in only one direction, indicating the morphology to be heterogeneous.

Figure 12 shows the molecular mechanism breakage points for the 10C_1000_ and 20C_1000_ chain models are more extended (ca. 250–300% deformation) than that for the 15C_1000_ chain model, where the 15C_1000_ model displays a sharp break point near 100% deformation. The higher breakage points in the 10C_1000_ and 20C_1000_ chain models could be attributed to the loosely packed crystallites. This can also be corroborated in the previous observation by Ramos et al., which shows that the intermolecular interaction radial distribution function (RDF) analysis of the PYS force-field models supports the tightly packed polymer chains more than their counterpart models obtained from Trappe force fields [14]. Despite low crystallinity and smaller lamellar stem lengths, the SSC computed in this work is valid with a true HDPE. Moreover, a slightly lower degree of crystallinity in Trappe models could explain the elongated break point. As a result, a stiffer elastic regime close to the range of 100–120 MPa is obtained, slightly consistent with the experimental values for PE [53]. This is because the rotational motion/sliding of the crystalline lamellar chains in the crystalline domains persists to a higher strain until it fully orients along the direction of tensile deformation, which is subsequently accompanied by a modulus yield. As evident, all-atom and united-atom model studies on the HDPE material simulations with a yield point of around 100–120 MPa have frequently appeared in MD simulation tensile tests [54,55]. In addition, we also affirm that our semi-crystalline models exhibit less pronounced double yield points, with a higher modulus, observed in the 10-, 15-, and 20-chain models, around a deformation strain of 10%, which is in line with the experiments and a comprehensive computational study by Dong et al. [20]. Hence, the consistency of the results supports the methodology applied for the model building and its characterization. In the extrapolation of the discussion for the inelastic regime, a slight strain hardening is seen for 20C_1000_ after the high yield point. Furthermore, we found a cavitation mechanism within 300% in the amorphous region, indicating that randomly ordered PE chains are the stress-holding atoms. The cavitation mechanism is well admitted for the HDPE materials, and these results align with previous studies, demonstrating that the faster strain rate model exhibits the cavitation mechanism. In contrast, the slower one reveals the melting or recrystallization mechanism [14,21,22,50]. Evidently, the strain rate-dependent comparative study on these models with semi-crystalline structures needs to be investigated to explore the strain rate-dependent deformation mechanism, which will be considered in the follow-up investigations.

## 4. Conclusions

This study proposes a unique way of preparing the HDPE models, starting from a single chain and then packing these chains in a way to obtain a semi-crystalline structure with an alternating lamellae orientation, which would be used to mimic the microstructure behavior undergoing mechanical recycling conditions. MD simulations are initiated to examine the preparation of a lamellar stacked semi-crystalline model using TraPPE force-field parameters on a minimalistic two-HDPE chain structure (C_1000_) model packed in rectangular and cubic boxes. Based on the anticipated results obtained for 2C_1000_ models, the 10-, 15-, and 20-chain models are replicated and characterized. The MD simulation results support the RC (2C_1000_) starting structure to prepare a lamellar stack-oriented theoretical HDPE model with microstructure characteristics such as crystalline stems and amorphous bridges, loops, and tail segments. Using this approach, the 10-chain (10C_1000_) model is the most reliable and computationally affordable model to test the mechanical recycling or desired applications of plastic materials—similar to the 15- and 20-chain models. However, it comes with a little more computational expense.

The structural characterization has revealed that the semi-crystalline network mimics alternate crystalline and amorphous states. Moreover, the order parameters show the semi-crystalline model with stacked lamellar orientation. The mass density profile along the *z*-axis of the simulation box is consistent with the crystalline state density of 1.0 g/cm^−3^ and amorphous state density of 0.85 g/cm^−3^, and most importantly, the total density of the semi-crystalline form displayed ~0.90–0.94 g/cm^−3^, which is consistent with the computational model studied earlier. In contrast, the degree of crystallinity proves the marginal heterogeneous lamellar orientations appeared along the *y*-axis in 20C_1000_, and the lamellar stem length marginally reduced as the number of chains increased from 15 (~95.5 Å) to 20 chains (~78.5 Å). Moreover, the degree of crystallinity further supports the orientation of the lamellar structures in the model. It segregates the amorphous and crystalline regions to visualize alternate lamellar orientations aligning the axis’s density profile.

The model behavior under uniaxial deformation is assessed under the applied strain of 10^10^ s^−1^. The findings showed that 10C_1000_, 15C_1000_, and 20C_1000_ models possessed elastic deformation, yield, and plastic flow at realistic atmosphere conditions (300 K and 0 atm), which further affirmed the formation of alternating lamella and amorphous phases in the models. The tensile deformation mechanism is attributed to the formation of cavitation in the amorphous region. The lamellar stems in the crystalline areas are aligned in the direction of deformation, consistent with previously established models [17,21,22,23,24,25,37,39,48]. The presented results demonstrate that the plastic material models can be easily prepared with our method and can be extrapolated to the bulk systems to understand the macroscopic properties at the molecular level.

## Figures and Tables

**Table 1:** TraPPE bonded and non-bonded force-field parameters for HDPE models are considered in this study.

TraPPE Force Field			
Bonded			
	Functional form	Parameters	
Bond potential	$E_{bond}=k_{b}(r-r_{0}{)}^{2}$	K_b_ (kcal/mol)	260.0
	Bond length	R_0_ (Å)	1.54
Angle potential	$E_{angle}=k_{b}(\theta -\theta_{0}{)}^{2}$	K_θ_ (kcal/mol/rad^2^)	62.1
	Bong angle	θ (Degree)	114.0
Dihedral potential	$E_{dihedral}=\sum_{0}^{n=3}C_{i}{(cos}^{i}\left(\phi \right)$)		
	Zeroth-order term	C_0_ (kcal/mol)	2.0
	First-order term	C_1_ (kcal/mol)	−4.01
	Second-order term	C_2_ (kcal/mol)	0.271
	Third-order term	C_3_ (kcal/mol)	6.29
Non-bonded			
van der Waals interactions	$E_{Lennard-Jones}=4\varepsilon_{ij}\{(\sigma_{ij}/r_{ij}{)}^{12}-(\sigma_{ij}/r_{ij}{)}^{6}\}$ $;\;\mathrm{where}\;\varepsilon_{ij}=\sqrt{\varepsilon_{i}\varepsilon_{j}};\;$ $\sigma_{ij}=\sqrt{\sigma_{i}\sigma_{j}}$		
	Depth of the potential energy	ε (kcal/mol)	0.091
	The distance at which the potential energy is zero	σ (Å)	3.95
	The cutoff distance beyond which interactions are neglected	Cut-off (Å)	14.0

**Table 2:** The mean number of kinks per chain and open-angle are the directly measured quantities of Z1+ code reported for all models. Three MD simulation conditions are considered: the equilibrated model at 450 K, the quenched model from 450 K to 300 K, and the isothermal crystallization at 300 K. The unit lengths are in Å.

Model	MD Condition	$\langle Z\rangle$
10C_1000_	1. Equilibrated model at 450 K	7.4
	2. Quenched model at 440 K	7.0
	3. Quenched model at 300 K	10.8
	4. Crystallized model at 300 K	10.0
15C_1000_	1. Equilibrated model at 450 K	7.2
	2. Quenched model at 440 K	8.3
	3. Quenched model at 300 K	13.4
	4. Crystallized model at 300 K	10.3
20C_1000_	1. Equilibrated model at 450 K	8.6
	2. Quenched model at 440 K	8.5
	3. Quenched model at 300 K	15.3
	4. Crystallized model at 300 K	11.3
Expt. [43]	HDPE Entanglement Density at 443 K	12.2

## Data Availability

The data supporting this study’s findings are available upon request from the corresponding author.

## Supplementary Materials

The following Supporting Information can be downloaded at: https://www.mdpi.com/article/10.3390/polym16020304/s1. Scheme S1. The step-by-step procedure for preparing lamellar stacked HDPE chain models. Table S1. Total density and the cell parameters of RC-2C_1000_ and its extended models of 10-chain, 15-chain, and 20-chain models in the initial (300 K)-NVT melted states (450 K), equilibrated state (450 K), quenched state (300 K), and isothermally cooled state at 300 K. All the densities are represented in g.cm^−3^, and cell parameters are in Å. Figure S1. A schematic representation of the Trappe forcefield-based 2C_1000_ HDPE semicrystalline model with lamella stacked orientation preparation. Figure S2. Potential energy decomposition for RC (2C_1000_) model at 450 K for the equilibration step representing the energy components: bond stretching, angle bending, dihedral torsion, van der Waals energy evolution over the MD simulation run for 10 ns (Δ*t* = 2 fs). Figure S3. The variation of the density in the sequential temperature quenching from 450 K to 400 K with 10 K per nanosecond time scale and from 400 K to 300 K with 5 K per 1 ns time length scales for the extended models. Figure S4. 10_C1000_ is a representative model to show all chains, individual chain dynamics, and ordering chains in the isothermal crystallization at 300 K. The models at 0 ns are amorphous and later evolve into crystalline lamellae as the simulations progress to 500 ns time length. Figure S5. The two-order parameters with >0.4 thresholds are computed for extended models, and the respective degree of crystallinity is highlighted in the box. The cyan color represents the crystal part, and the blue color represents the amorphous part of the HDPE polymer.
