# Supplementary material for: Preparation and Characterization of High-Density Polyethylene with Alternating Lamellar Stems Using Molecular Dynamics Simulations
Source: Polymers (Basel). 2024 Jan 22;16(2):304. doi: 10.3390/polym16020304 (PMC10819154; doi:10.3390/polym16020304)
Supplement: Supplementary file 1 [file polymers-16-00304-s001.zip › polymers-2829529-supplementary.pdf]

Supporting Information

Preparation and Characterization of High-Density Polyethylene with Alternating Lamellar Stem Using Molecular Dynamics Simulations

Mohammed Althaf Hussain[1,*], Takashi Yamamoto[2], Syed Farooq Adil[3], and Shigeru Yao[1,*]

[1]Central Research Institute, Fukuoka University, Fukuoka 814-0180, Japan

[2] Graduate School of Science and Engineering, Yamaguchi University, Yamaguchi 753-8512, Japan

[3] Department of Chemistry, College of Science, King Saud University, P.O. Box 2455, Riyadh 11451, Saudi Arabia

*Correspondence: altaf.mh7@gmail.com; shyao@fukuoka-u.ac.jp

Content	Pages
**Scheme S1.** The step-by-step procedure for preparing lamellar stacked HDPE chain models.	S2
**Table S1.** Total density and the cell parameters of RC-2C_{1000} and its extended models of 10-chain, 15-chain, and 20-chain models in the initial (300K)-NVT melted states (450K), equilibrated state (450K), quenched state (300K), and isothermally cooled state at 300K. All the densities are represented in g.cm$^{-3,}$ and cell parameters are in Å.	S3
**Figure S1.** A schematic representation of the Trappe forcefield-based 2C_{1000} HDPE semicrystalline model with lamella stacked orientation preparation.	S4
**Figure S2.** Potential energy decomposition for RC (2C_{1000}) model at 450K for the equilibration step representing the energy components: bond stretching, angle bending, dihedral torsion, van der Waals energy evolution over the MD simulation run for 10 ns ($\Delta t = 2\ fs$).	S5
**Figure S3.** The variation of the density in the sequential temperature quenching from 450K to 400K with 10K per nanosecond time scale and from 400K to 300K with 5K per 1 ns time length scales for the extended models.	S6
**Figure S4.** 10C_{1000} is a representative model to show all chains, individual chain dynamics, and ordering chains in the isothermal crystallization at 300K. The models at 0 ns are amorphous and later evolve into crystalline lamellae as the simulations progress to 500 ns time length.	S7
**Figure S5.** The two-order parameters with > 0.4 thresholds are computed for extended models, and the respective degree of crystallinity is highlighted in the box. The cyan color represents the crystal part, and the blue color represents the amorphous part of the HDPE polymer.	S8

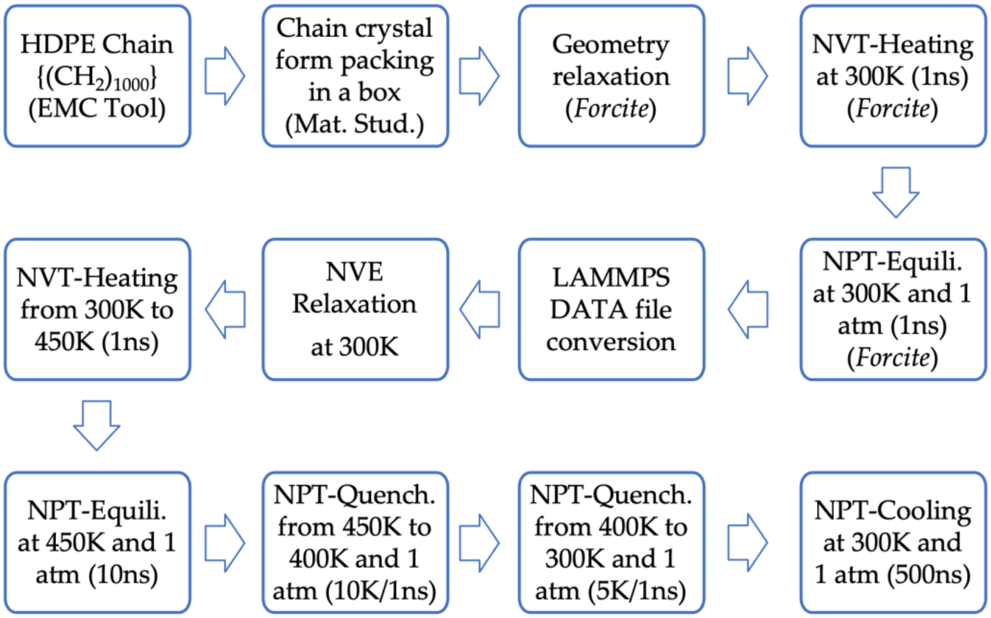

Scheme S1. The step-by-step procedure for preparing lamellar stacked HDPE chain models.

Table S1. Total density and the cell parameters of RC-2C_{1000} and its extended models of 10-chain, 15-chain, and 20-chain models in the initial (300K)-NVT melted states (450K), equilibrated state (450K), quenched state (300K), and isothermally cooled state at 300K. All the densities are represented in g.cm$^{-3,}$ and cell parameters are in Å.

**Conditions**	**Trappe Force field parameters**							
	**RC-2C_{1000}**		**10C_{1000}**		**15C_{1000}**		**20C_{1000}**	
	ρ	Cell (a,b,c)	ρ	Cell	ρ	Cell	ρ	Cell
Input and NVT melting at 450K	*1.184*	a= 25.057	*0.931*	50.0	*0.239*	90.0	*0.922*	51.266
		b=25.057		50.0		90.0		153.798
		c=62.642		100.0		180.0		64.082
NPT Equilibration at 450K	*0.748*	a=29.204	*0.758*	53.534	*0.749*	61.533	*0.755*	54.790
		b=29.204		53.534		61.533		164.370
		c=73.010		107.068		123.066		68.487
Quenched model at 300K	*0.859*	*a=27.888*	*0.847*	51.601	*0.854*	58.909	*0.851*	52.648
		b=27.888		51.601		58.909		157.942
		c=69.721		103.202		117.817		65.809
Crystallized model at 300K	*0.941*	a=27.054	*0.911*	50.360	*0.909*	57.676	*0.900*	51.662
		b=27.054		50.360		57.676		154.987
		c=67.635		100.720		115.353		64.578

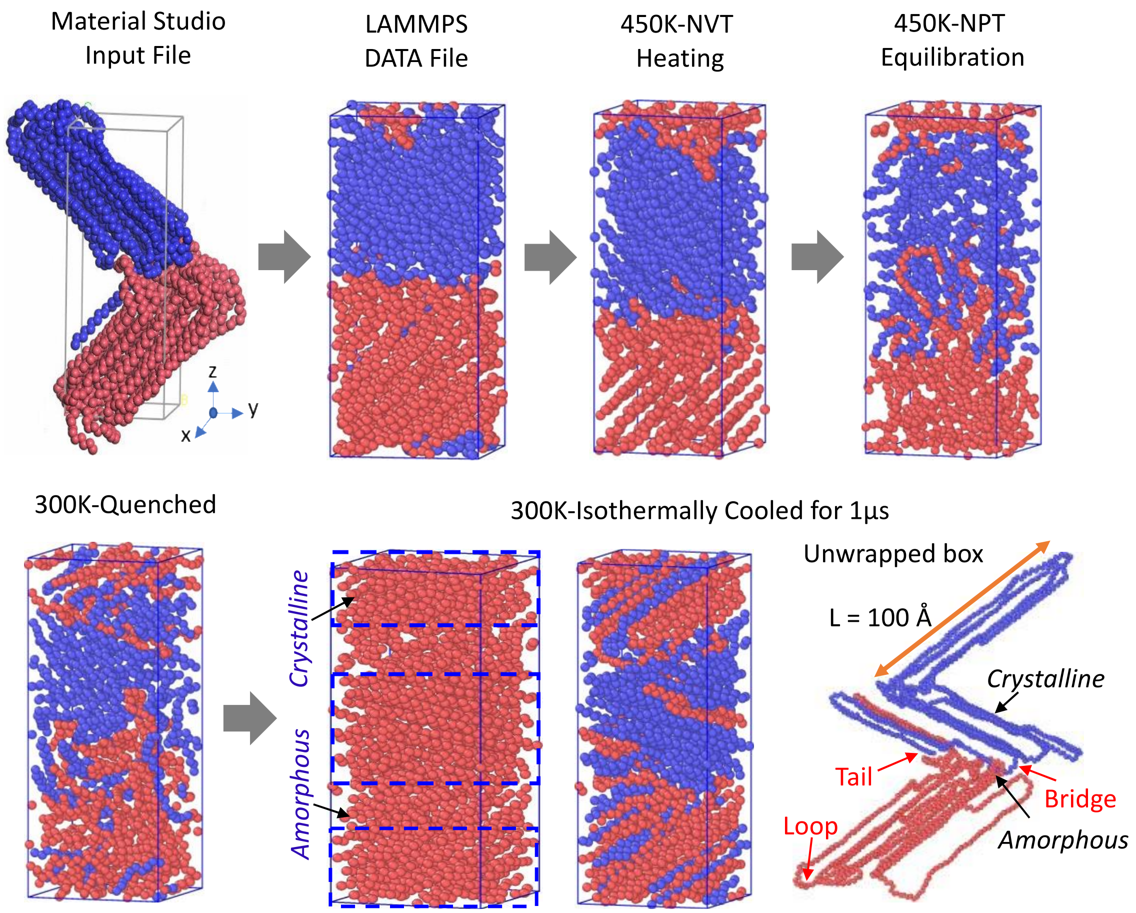

Figure S1. A schematic representation of the Trappe forcefield-based $2C_{1000}$ HDPE semicrystalline model with lamella stacked orientation preparation.

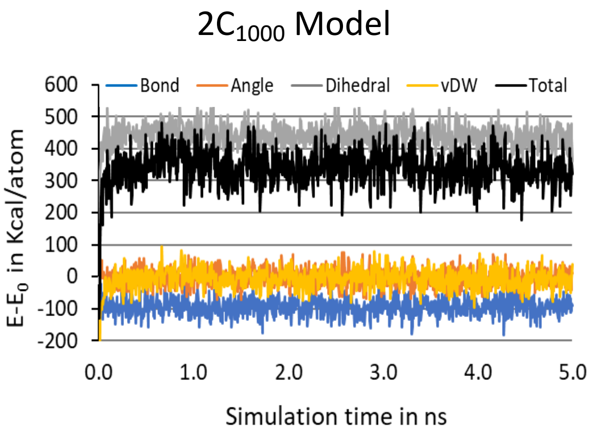

Figure S2. Potential energy decomposition for RC ($2C_{1000}$) model at 450K for the equilibration step representing the energy components: bond stretching, angle bending, dihedral torsion, van der Waals energy evolution over the MD simulation run for 10 ns ($\Delta t = 2\ fs$).

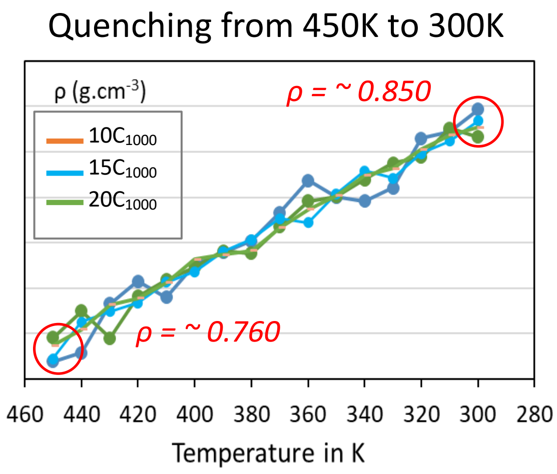

Figure S3. The variation of the density in the sequential temperature quenching from 450K to 400K with 10K per nanosecond time scale and from 400K to 300K with 5K per 1 ns time length scales for the extended models.

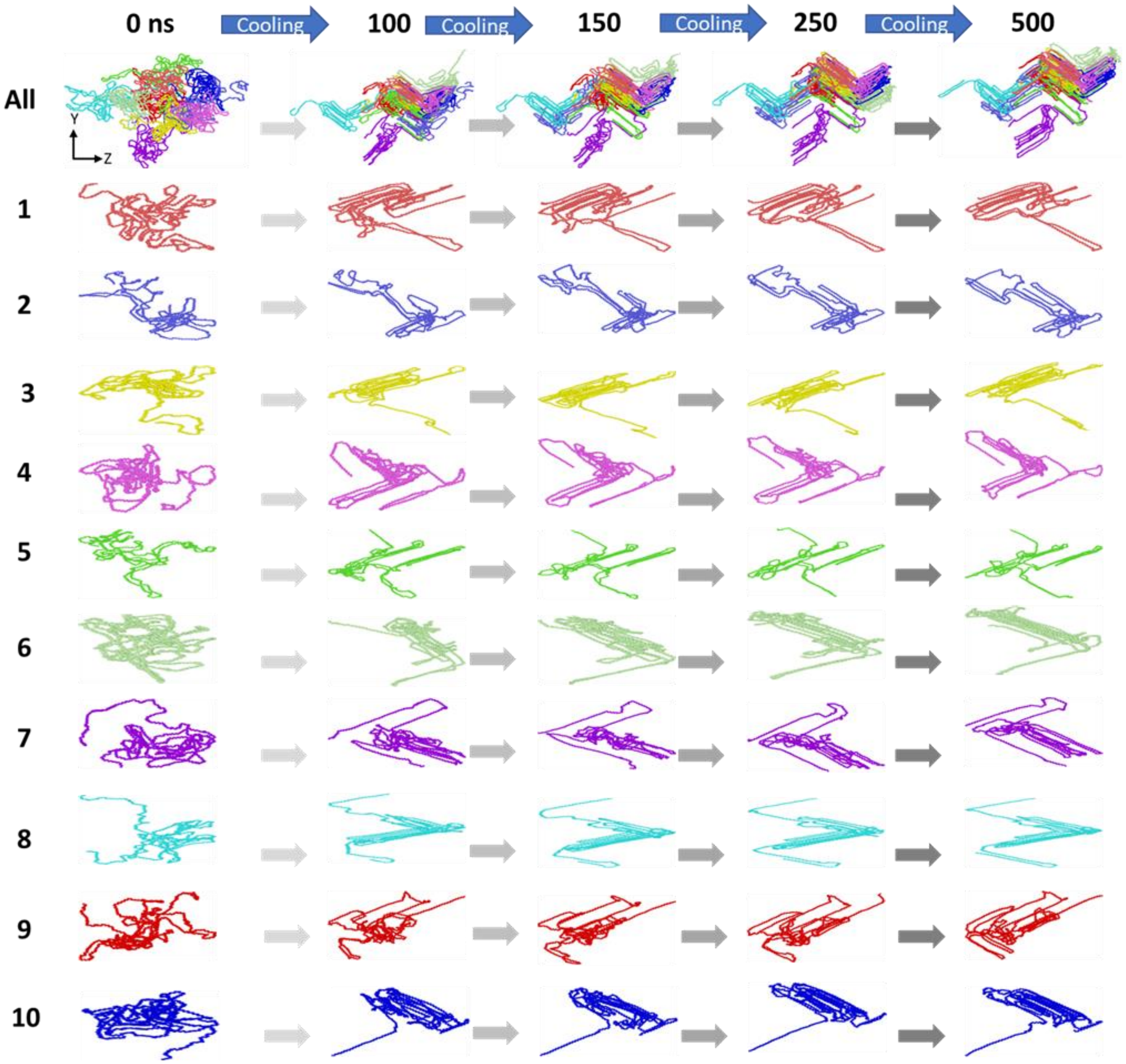

Figure S4. $10C_{1000}$ is a representative model to show all chains, individual chain dynamics, and ordering chains in the isothermal crystallization at 300K. The models at 0 ns are amorphous and later evolve into crystalline lamellae as the simulations progress to 500 ns time length.

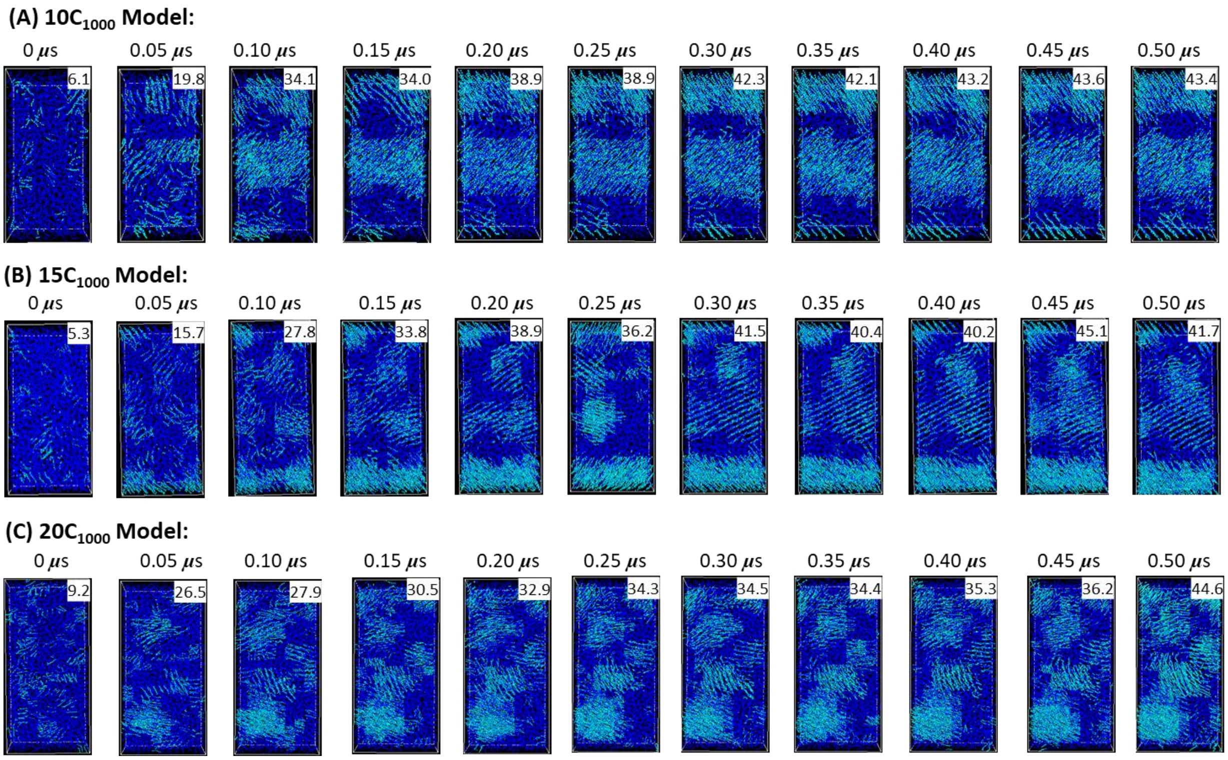

Figure S5. The two-order parameters with > 0.4 thresholds are computed for extended models, and the respective degree of crystallinity is highlighted in the box. The cyan color represents the crystal part, and the blue color represents the amorphous part of the HDPE polymer.
